# A proof of concept study for the differentiation of SARS-CoV-2, hCoV-NL63, and IAV-H1N1 in vitro cultures using ion mobility spectrometry

**DOI:** 10.1038/s41598-021-99742-7

**Published:** 2021-10-11

**Authors:** M. Feuerherd, A.-K. Sippel, J. Erber, J. I. Baumbach, R. M. Schmid, U. Protzer, F. Voit, C. D. Spinner

**Affiliations:** 1 Institute of Virology, School of Medicine, Technical University of Munich, 81675 Munich, Germany; 2 Institute of Virology, Helmholtz Zentrum München, Munich, Germany; 3 B. Braun Melsungen AG, Branch Dortmund, Center of Competence Breath Analysis, BioMedicalCenter, Dortmund, Germany; 4 Department of Internal Medicine II, University Hospital Rechts Der Isar, School of Medicine, Technical University of Munich, Munich, Germany; 5 German Center for Infection Research (DZIF), Munich Partner Site, Munich, Germany

**Keywords:** Analytical chemistry, Viral infection, Predictive markers, Biomarkers

## Abstract

Rapid, high-throughput diagnostic tests are essential to decelerate the spread of the novel coronavirus disease 2019 (COVID-19) pandemic. While RT-PCR tests performed in centralized laboratories remain the gold standard, rapid point-of-care antigen tests might provide faster results. However, they are associated with markedly reduced sensitivity. Bedside breath gas analysis of volatile organic compounds detected by ion mobility spectrometry (IMS) may enable a quick and sensitive point-of-care testing alternative. In this proof-of-concept study, we investigated whether gas analysis by IMS can discriminate severe acute respiratory syndrome coronavirus 2 (SARS-CoV-2) from other respiratory viruses in an experimental set-up. Repeated gas analyses of air samples collected from the headspace of virus-infected in vitro cultures were performed for 5 days. A three-step decision tree using the intensities of four spectrometry peaks correlating to unidentified volatile organic compounds allowed the correct classification of SARS-CoV-2, human coronavirus-NL63, and influenza A virus H1N1 without misassignment when the calculation was performed with data 3 days post infection. The forward selection assignment model allowed the identification of SARS-CoV-2 with high sensitivity and specificity, with only one of 231 measurements (0.43%) being misclassified. Thus, volatile organic compound analysis by IMS allows highly accurate differentiation of SARS-CoV-2 from other respiratory viruses in an experimental set-up, supporting further research and evaluation in clinical studies.

## Introduction

Severe acute respiratory syndrome coronavirus 2 (SARS-CoV-2) infections have spread rapidly around the world, causing coronavirus disease 2019 (COVID-19), which has emerged as a deadly pandemic^1^. Fast and high-throughput diagnostic testing is essential to combat the pandemic and disrupt transmission chains and disease spread. Currently, the diagnosis of acute SARS-CoV-2 infection mainly relies on RT-PCR performed on nasopharyngeal swabs^2^. However, long turnaround times associated with RT-PCR testing in centralized laboratories and the need for fully trained staff and dedicated PCR equipment substantially limit the effectiveness of isolation measures and contact tracing. In the emergency department setting, delays can lead to poor patient flow and nosocomial infections^3^. Furthermore, widespread PCR testing is increasingly hampered by a global shortage of PCR reagents^2^ and the high costs are particularly challenging for developing countries^4^. The much simpler point-of-care lateral flow antigen assay offers results within 30 min^5^. However, a major disadvantage is the significantly lower sensitivity of rapid antigen tests than that of RT-PCR, especially at low viral loads or when performed a few days after the start of symptoms, when hospital admission is required^6–8^.

Alternative cost-effective diagnostic tests that can be performed without specific laboratory personnel or reagents and allow rapid testing at the point-of-care are urgently needed. Breath gas analysis of volatile organic compounds (VOCs) detected by ion mobility spectrometry (IMS) is a robust, fast, and simple analysis method that can fulfill all the requirements mentioned above.

For a detailed review of the principles of MCC-IMS, please see Cumeras et al.^9,10^. In short, in IMS, the analytes are ionized and accelerated along an electric field in a drift tube. For improved sample separation, a so-called multi-capillary column (MCC) can be connected upstream. Thus, analytes can be distinguished by IMS for their drift time in the drift chamber and by MCC for their retention time in the multi-capillary column, and signal intensity. For a more detailed description, please refer to the supplementary information. Compared to mass spectrometry, MCC-IMS has distinct advantages for breath gas analysis: no vacuum is required, humidity does not disturb the analysis, and devices are lighter and cheaper. The short measurement time of about 5–12 min for MCC-IMS is another benefit in contrast to a thermodesorption-based mass spectrometry method for the analysis of VOCs like GC/MSD (gaschromatography coupled mass selective detector), which takes 30–120 min. The latter method additionally relies on high-purity helium and liquid nitrogen for the thermodesorption^11^.

In recent years, different methods of breath analysis have been increasingly applied for the detection of viruses and bacteria, such as rhinovirus^12^, influenza viruses^13,14^ and various bacteria^15^. The first efforts to detect SARS-CoV-2 by breath gas analysis have been reported^16–20^, but further refinement of methods and studies with higher patient numbers are urgently needed.

In this proof-of-concept study, we aimed to investigate whether gas analysis by MCC-IMS allows the discrimination of SARS-CoV-2 from other respiratory viruses in an experimental setup. We performed repeated gas analyses of air samples collected from the headspace of virus-infected in vitro cell cultures over a period of up to 5 days. We hypothesized that differences in the peak intensity of IMS-chromatograms would allow model building to differentiate between human respiratory viruses. If successful, the methods could find their way in clinical applications in the form of breath gas analysis.

## Results

### VOC analysis by MCC-IMS of air retrieved from the headspace of virus-infected in vitro cultures

**Figure 1:**
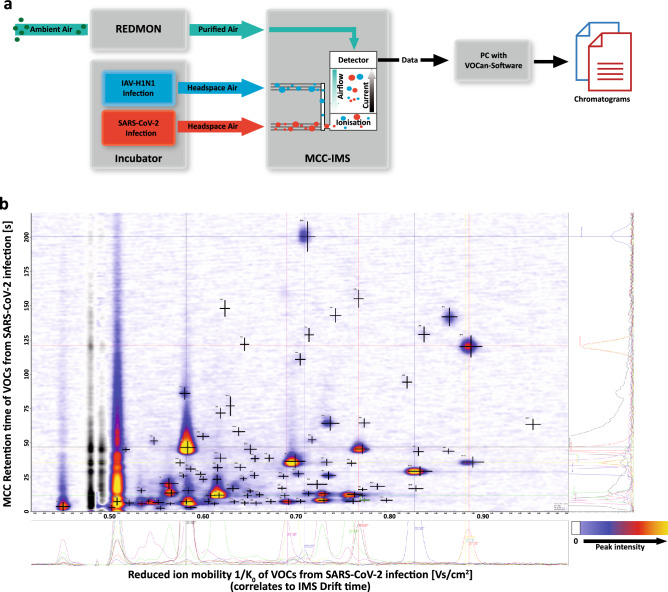
Separation and analysis of headspace air samples of SARS-CoV-2 and other respiratory virus cultures by airflow through a multi-capillary column (MCC), followed by ion mobility spectroscopy (IMS). (**a**) Schematic depiction of the experimental setup. Ambient air was purified by a Redmon device and supplied to the MCC-IMS. Headspace air samples (10 mL) of SARS-CoV-2, hCoV-NL63 (not shown), or IAV-H1N1 cultivated in pairs in vitro in a 37 °C, 5% CO_2_ incubator were collected every 12.5 min and transferred to the MCC-IMS. Samples were separated according to their retention time in the MCC and their drift time in IMS. Data were analyzed using the *VOCan* software generating IMS-chromatograms of the samples. The scheme was generated with Affinity Designer 1.10 (https://affinity.serif.com/en-us/designer/). (**b**) Exemplary IMS-chromatogram of a 10 mL headspace air sample collected after SARS-CoV-2 infection. In total, 93 different peaks were identified manually and with an automated run of established parameters (with the software VisualNow version 3.7). The positions of the peaks are marked by black crosses (**+**). Individual spectra for prominent peaks are shown below the chromatogram, and total ion current lines for the peak positions are shown on the right. The chromatogram was produced with VisualNow version 3.7 (permission granted as provided to the editor) and the axis labeling and legend were added with Affinity Designer 1.10 (https://affinity.serif.com/en-us/designer/).

To perform an in-depth analysis of VOCs (volatile organic compounds; please note: not variant of concern) emitted from virus-infected in vitro cultures, culture flasks were connected to an MCC-IMS instrument, as described in the “Methods” section and visualized in Fig. 1a. To control for virus-independent effects on the signals detected, measurements of blank flasks, flasks filled with medium only and flasks with uninfected cultured cells were performed. VeroE6 cells were infected with SARS-CoV-2, human coronavirus-NL63 (hCoV-NL63), or influenza A virus H1N1 (IAV-H1N1), and MCC-IMS was performed every 12.5 min for 72 h of cultivation. Peaks in the three-dimensional IMS-chromatograms, which correspond to VOCs, were identified manually (V01–V93), with peak intensity generally correlating with VOC concentration. Repeated MCC-IMS measurements revealed a broad spectrum of peaks, many of which were detected in infection cultures of all viruses. We did not detect any peak, which was present only in a single or in only two viral infections. An example of an IMS-chromatogram of SARS-CoV-2 infection is shown in Fig. 1b. These analyses showed that MCC-IMS can be used to detect VOCs in the headspace air of SARS-CoV-2 in vitro.

### Longitudinal MCC-IMS measurements revealed improved peak differentiation on day 3 post infection

To observe the effects of ongoing viral replication on VOCs collected from the headspace of tissue cultures, longitudinal MCC-IMS measurements for SARS-CoV-2, hCoV-NL63, and IAV-H1N1 were performed. In every experimental setup, two different virus groups were sampled in parallel with alternating measurements and blank measurements every 12.5 min. The experiments ran for up to a maximum time of 113 h.

**Figure 2:**
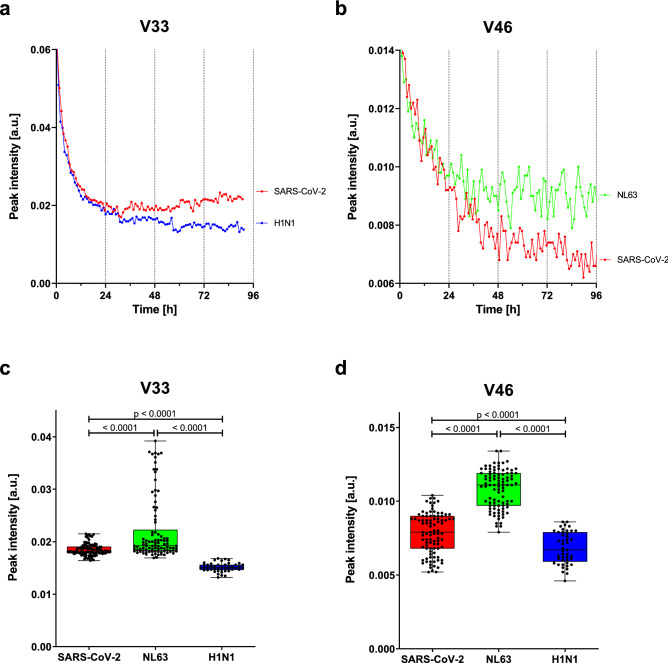
Peak intensities for SARS-CoV-2, human coronavirus NL63, and influenza A virus H1N1 show progression over time and differ between the viruses. Signal intensities in arbitrary units (a.u.) of peaks (**a**) V33 and (**b**) V46 from headspace air samples of a single experimental setup of in vitro cultures of SARS-CoV-2 (red), IAV-H1N1 (blue), and hCoV-NL63 (green) are shown over 96 h post infection (p.i.). Box-and-whisker plots represent the signal intensity at day 3 p.i. of peak (**c**) V33, which had the lowest Norm U-value in the Mann–Whitney U-test (see Supplementary Table S1) with SARS versus NL63: 0.277, SARS versus H1N1: 0.002 and NL63 versus H1N1: 0.000. The same is shown for (**d**) V46, which showed U-values of SARS versus NL63: 0.059, SARS versus H1N1: 0.290 and NL63 versus H1N1: 0.006. The intensity of every single measurement is indicated by a black dot. Central lines show the median, with colored boxes indicating interquartile ranges. The *p* values were calculated with Mann–Whitney U test and are shown for the comparison of data from day 3. Data was visualized with GraphPad Prism 9.2.0 (https://www.graphpad.com/).

The intensity of some distinct peaks increased over time, while it decreased for others, likely resembling changes in cell metabolism after viral infection. For peaks that differed in intensity between infections at late time points, we found that the intensities often started out at similar levels and began to diverge only on or after day 2 post infection. As an example, the peaks V33 (between SARS-CoV-2 and IAV H1N1 infection) and V46 (between SARS-CoV-2 and hCoV-NL63 infection) showed markedly different intensities between the indicated infections at approximately 48 h post infection (Fig. 2a, b). These findings suggest that the differentiation of viruses using VOCs would be most promising on day 3 post infection in this preclinical model.

From 232 longitudinal experiments (SARS-CoV-2, *n* = 93; hCoV-NL63, *n* = 94; IAV-H1N1, *n* = 45), all measurements performed from 49 to 72 h post infection were considered for further peak analysis. For paired comparison of single peaks, low-rank sums computed by Mann–Whitney U testing signify a high potential to discriminate between the viruses. Peak analysis revealed nine peaks with a rank sum of < 0.2 for SARS-CoV-2 versus hCoV-NL63, 11 peaks for SARS-CoV-2 versus IAV-H1N1, and 20 peaks for hCoV-NL63 versus IAV-H1N1. MCC-IMS does not allow direct identification of molecules, but by comparing the peaks to a database of IMS-chromatograms of described chemical compounds, it was possible to assign specific molecules to each peak according to their drift and retention times. For some peaks, more than one molecule fitted the parameters (Supplementary Table S1 lists up to three molecules in descending probability for the five peaks with the lowest rank sums for each virus). Most prominently, peaks with high differentiating potential matched those of pentanal (peak V33), 2-butanone (peak V33), and nonane (peak V57) from an IMS-chromatogram database.

Signal intensities of all day 3 measurements for SARS-CoV-2, hCoV-NL63, and IAV-H1N1 of peaks V33 and V46, which are the peaks with the lowest norm U score in the paired comparison, are depicted in Fig. 2c,d. The signal intensity for the V33 peak was substantially lower for IAV-H1N1 than for the two coronaviruses. However, hCoV-NL63 had a significantly higher peak for V46 signal intensity than SARS-CoV-2 and IAV-H1N1 did. Data for other peaks used for virus differentiation are shown in Supplementary Fig. S2. Taken together, we were able to identify peaks whose intensities differed in the individual virus groups.

### Decision trees and forward selection allow highly accurate differentiation of SARS-CoV-2 from human coronavirus NL63 and influenza A virus H1N1

As described above, we found large differences in the signal intensities of individual peaks in the different virus groups. Therefore, we wanted to investigate whether by combining certain peaks, a model allowing the assignment of a given sample to one of the three virus groups could be generated.

**Figure 3:**
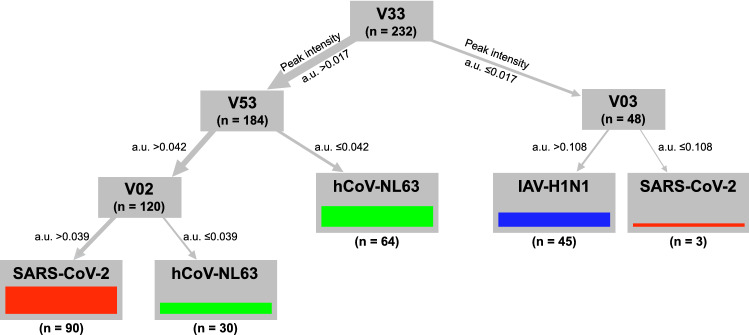
Three-step decision tree of day 3 measurements classifies SARS-CoV-2, human coronavirus NL63, and influenza A virus H1N1 correctly without misassignment. Group assignment was conducted from top to bottom. Every grey box shows an MCC-IMS chromatogram peak, which was used in the decision tree and the number (n) of measurements. Numbers on grey arrows indicate peak intensity in arbitrary units (a.u.) of the peak above. The thickness of the arrows pointing from one box to the next shows the relative number of measurements with the respective peak intensity. The height of colored bars indicates the number of measurements classified in the respective group. Colored bars indicate the samples of the respective virus group classified, with red representing SARS-CoV-2; green, hCoV-NL63; and blue, IAV-H1N1. Note that all columns consisting of only one color indicate no wrong assignment. The graphic was visualized with Affinity Designer 1.10 (https://affinity.serif.com/en-us/designer/).

Decision trees represent an established, clear, and easy-to-understand assignment model that results in increased sensitivity and specificity of diagnostic measures (see “Methods”). Figure 3 shows a decision tree generated with data from day 3 with the specification to use the least number of variables to differentiate between the viruses (Supplementary Table S3-S6 list statistical parameters showing the differentiation power of peaks V33, V02, V03 and V53 alone). Remarkably, only four peaks and a maximum of three differentiation steps were sufficient to assign all measurements to the correct set of experiments with a certain virus, with no single measurement misclassified.

Decision trees are impressive in their simplicity but tend to become confusing with large data sets and are prone to overfitting^21,22^. A forward selection approach, which is a variant of stepwise regression, is more suitable for assignment model building. Starting with an empty model, one variable is added after the other, starting with the variable with the highest differentiation power, until the model cannot be improved by adding more variables. Our methodology on this is described in the “Methods” section. We provided the algorithm with data of day 3 post-infection. The forward selection used the peaks V04, V21, V29, V30, V34, V39, V46, V61, and V93 (Table 1; please refer to Supplementary Table S1 for more information on the peaks and to Supplemental Fig. S2 for data regarding the peak intensities), which resulted in a sensitivity of 98.9% for SARS-CoV-2 and 100% for hCoV-NL63 and IAV-H1N1, with only 1 of 231 misclassified measurements. The specificity was 100% for the identification of SARS-CoV-2, 99.3% for hCoV-NL63, and 100% for IAV-H1N1. Upon including data from all measurement days, the forward selection approach resulted in a sensitivity of 87.4% for SARS-CoV-2, 87.4% for hCoV-NL63, and 89.4% for IAV-H1N1 (see Supplementary Table S7). The specificity values were 88.2%, 97.0%, and 95.3% for SARS-CoV-2, hCoV-NL63, and IAV-H1N1, respectively. This supports the use of algorithms like decision trees and forward selection to allow precise identification of respiratory viruses by breath gas analysis using MCC-IMS.

**Table 1:** Forward selection allows highly accurate classification of SARS-CoV-2, human coronavirus NL63, and influenza A virus H1N1.

		**Infection**			Total	Positive predictive value	Negative predictive value
		SARS-CoV-2	hCoV-NL63	IAV-H1N1			
**Test**	SARS-CoV-2	92	0	0	92	100.0%	100.0%
	hCoV-NL63	1	94	0	95	98.9%	99.3%
	IAV-H1N1	0	0	45	45	100.0%	100.0%
	Total	93	94	45			
	Sensitivity	98.9%	100.0%	100.0%			
	Specificity	100.0%	99.3%	100.0%			

Day 3 post infection headspace air samples of infections were evaluated with forward selection to determine the peaks. Peaks chosen for the forward selection are V04, V21, V29, V30, V34, V39, V46, V61, and V93.

## Discussion

We found that MCC-IMS allowed reproducible analysis of VOCs whose peak intensities changed upon infection of cell cultures with respiratory viruses. Characteristic peak intensities for different virus infections enabled computer-guided differentiation of SARS-CoV-2, seasonal hCoV-NL63, and influenza virus IAV-H1N1 with high precision.

Rapid, high-throughput diagnostic testing is indispensable to constrain the spread of the COVID-19 pandemic. Breath gas analysis by MCC-IMS is a promising new method for the rapid detection of respiratory tract infections at the point of care^19,23^. In this proof-of-concept study, we established a preclinical model system to perform in-depth VOC analysis using MCC-IMS of SARS-CoV-2 and other respiratory viruses. Air collected from the headspace of virus-infected in vitro cultures was analyzed continuously over the course of up to 5 days, and more than 100 different peaks were detected in the IMS-chromatogram for SARS-CoV-2, hCoV-NL63 (another human coronavirus), and IAV-H1N1. Changes in the peak intensity over the first three days post infection were observed in a virus-specific manner, likely reflecting the influence of viral replication on cell metabolism. Therefore, the data imply that similar changes could occur in the human respiratory epithelium and could be detected by VOC analysis of breath from patients with respiratory infection.

By comparing the IMS-chromatograms with a database of chromatograms of described chemical substances, the peaks differentiating the viruses could be assigned to organic molecules such as pentanal, 2-butanone, and nonane, which have previously been identified in association with pulmonary disease^15,24–26^.

Due to the kinetic changes in peak intensity, as described in the Results section, the differentiation of the viruses was most robust on day 3 post infection. We hypothesize that the biological explanation for this observation is that a stable viral replication must be first established and only then leads to a release of specific volatile organic compounds to the headspace air. At late infection time points, non-infection-related apoptosis likely plays a role. Therefore, taking data from all 5 days of measurement resulted in decreased sensitivity and specificity. Considering the peak intensity of the four peaks, the three viruses (SARS-CoV-2, hCoV-NL63, and IAV-H1N1) could be classified correctly in a three-step decision tree without misallocation. Using an assignment model based on forward selection, SARS-CoV-2 infection headspace air samples were detected with a sensitivity of 98.9% and a specificity of 100.0%.

The results of this study suggest that VOC analysis by MCC-IMS allows for robust differentiation of SARS-CoV-2 from other respiratory viruses in this preclinical model. By identifying SARS-CoV-2 infection-specific changes to VOCs by MCC-IMS breath gas analysis, Ruszkiewicz et al*.* provided the first evidence that distinguishing SARS-CoV-2 infection from other respiratory disorders might be possible in the clinic^17^. Further research with a clinical validation study is needed to demonstrate the feasibility of breath analysis using MCC-IMS as a diagnostic tool for the rapid detection of SARS-CoV-2 in routine clinical care and point-of-care environments. Differential breath gas analysis of upper and lower respiratory tract samples would be of particular interest to gain a better understanding of the time course and severity of COVID-19. MCC-IMS technology combined with an automated analysis algorithm could provide results within 10 min, but it remains to be shown whether a SARS-CoV-2-specific signal can be detected despite background noise of possible other viral or bacterial infections or colonizations, comorbidities, smoking, or food intake.

## Methods

### Cultivation process

Vero cells are African green monkey epithelial kidney cells, isolated in Japan in 1962^27^. Vero cells are widely used for virus production and the VeroE6 clone was cultivated to inhibit growth upon contact to allow for more efficient virus propagation^28,29^. VeroE6 cells were obtained from ATCC, Manassas, Virginia, US (Ref CRL-1586). VeroE6 cells were cultivated in Dulbecco's Modified Eagle Medium (DMEM) (Gibco, Thermo Fisher Scientific, Carlsbad, CA, USA) with 5% FCS (fetal calf serum), 1% P/S (penicillin / streptomycin), 1% l-Glut (L-glutamine), 1% NEAA (non-essential aminoacids), and 1% sodium pyruvate (all Gibco) at 37 °C and 5% CO_2_ (carbon dioxide). Cells were infected in T-75 flasks (TPP, Trasadingen, Switzerland) with the SARS-CoV-2 strain hCoV-19/Germany/BAV-PL-virotum-nacq/2020 (GISAID accession ID: EPI_ISL_582134) at a multiplicity of infection (MOI) of one infectious virus particle per cell. Infection with human coronavirus-NL63 or influenza A virus H1N1 strain A/WSN/1933 was performed at an MOI of 1. All infection procedures were performed in cell culture fume hoods of the BSL-3 facility at the Institute of Virology, Technical University of Munich, after obtaining approval from the local regulatory officials at the General Administration of the Free State of Bavaria, Munich, Germany.

### Sample preparation and sampling procedure

After ultraviolet germicidal irradiation, T-75 flasks were equipped with an adapter attached to an M5 screw thread drill hole on the top side of the flask. T-75 test flasks were either filled with 12 mL of DMEM or used for the cultivation of VeroE6 cells and then transferred to the BSL-3 facility. Blank flasks, flasks with medium or with cultured cells, but without infection were used as controls. MCC-IMS spectra of controls and virus infections are shown in Supplementary Figs. S8 and S9. Test flasks were kept at 37 °C and 5% CO_2_ during the course of the experiment for at least 72 h.

PTFE tubes (3.2 mm, B. Braun Melsungen AG, Center of Competence Breath Analysis, formerly B & S Analytik GmbH, Dortmund, Germany) were used to connect the test flasks to an MCC-IMS instrument. Air samples (60 mL) were taken every 12.5 min for 10 s from the inside of the test flasks and for blank measurements without allowing any backflow of the sampled gas to the experimental flask and 10 mL of the air were transferred to the gas analysis.

### Gas analysis using MCC-IMS

Gas analysis was performed using an MCC-IMS system (Breath Discovery; B. Braun Melsungen AG, Center of Competence Breath Analysis, Dortmund, Germany). Minor modifications of the instrument allowed continued alternating gas analysis of the headspace of the two test flasks connected to it. From each unit, 60 mL of headspace was sampled of which 10 mL were transferred to the MCC (type OV-5; Multichrom Ltd., Moscow/Novosibirsk, Russia). Pre-separation of VOCs was performed using an MCC equipped with 1,000 capillaries in parallel (inner diameter 40 µm, film thickness 200 nm), followed by a second separation by IMS.

For IMS, a 95 MBq ^63^Ni ß-radiation source was used to ionize a carrier gas (purified room air provided by the instrument REDMON (B. Braun Melsungen AG, Center of Competence Breath Analysis, Dortmund, Germany), which in turn ionized the sample by ion–molecule reactions. The ionized analytes were detected on a Faraday plate at the end of the drift tube of the IMS by direct measurement of the charge.

As an internal experimental control, we used the reactant ion peak (RIP) technology^30^. This method was discussed and established for a long time for its usage in IMS^31^. The idea is that protonated water molecules would show a constant signal in IMS when using a molecular sieves trap to keep the moisture level of the purified carrier gas constant. While the chemical characteristics of the protonated water peak (corresponding to the RIP) would not change, the amount is only influenced by the temperature, which can also be controlled in the present setting.^32^ Every other compound can therefore be evaluated relative to the constant RIP.

The VOCan software (B.Braun Melsungen AG, Center of Competence Breath Analysis, Dortmund, Germany) was used to control the measurements, collect the data and record the three-dimensional IMS chromatogram by the detection of the drift time, retention time and signal intensity. VOCs were then identified using IMS-chromatograms and were described by their corresponding drift time (via IMS), retention time (via MCC), and signal intensity, which indicate the relative concentration of the analyte^33^. The software VisualNow version 3.7 (B. Braun Melsungen AG, Center of Competence Breath Analysis, Dortmund, Germany) was used for manual peak identification (for further details regarding peak findings, please see Supplementary Table S10).

### Peak analysis

Using the software VisualNow version 3.7 (B. Braun Melsungen AG, Center of Competence Breath Analysis, Dortmund, Germany), 93 different peaks were identified by manual supervision. Automated runs were performed using the parameters listed in Supplementary Table S10. The exact peak positions can be provided upon request.

Identified peaks were consecutively numbered from V01 to V93 (referring to virus infection compounds 1 through 93), in such a manner that the peak number did not refer to a single infection but could be shared between infection experiments with different viruses.

Chemical substances corresponding to peaks in the IMS-chromatogram were identified by comparison with chromatograms of known substances using the database 20160426_SubstanzDbNIST_122_St_layer (B. Braun Melsungen AG, Center of Competence Breath Analysis, Dortmund, Germany), which can be provided upon request as described in the section regarding Data availability. Here, the smallest distance within the IMS-chromatogram between the peaks of interest was taken in comparison to the peaks in the database with respect to drift time, retention time, and visual control (using VisualNow).

### Decision tree

A decision tree is a decision support tool that uses a tree-like model of decisions. Each internal node represents a test of a feature, with each branch representing one of the possible test results and each leaf node represents a classification^34^.

The decision trees in this project were created with a Chi-square Automatic Interaction Detectors (CHAID)-based algorithm (JIBB_DT_1206026 and JIBB_ROC_120624; further details provided upon request) using the program RapidMiner Studio version 9.0 (Rapid Miner, Boston, MA, USA).

### Forward selection

Stepwise regression is a method of fitting regression models in which the choice of predictive variables is carried out by an automatic procedure. Main approaches of stepwise selection are the forward selection, backward elimination and a combination of the two. Forward selection starts with no variables in the model and tests the addition of each variable using a chosen model fit criterion. The variable, whose inclusion gives the most statistically significant improvement of the fit, is added and the process is repeated until no improvement to the model can be achieved^35^. The software RapidMiner Studio version 9.0 (Rapid Miner, Boston, MA, USA) was used for the calculation of forward selection.

### Visualization

Diagrams were prepared using GraphPad Prism 9.2.0 (GraphPad, La Jolla, CA, USA), and figures were designed in Affinity Designer 1.10 (Serif (Europe) Ltd, West Bridgford, UK).

### Statistical analyses

Statistical analyses were performed using GraphPad Prism software version 9.2.0 (GraphPad, La Jolla, CA, USA) and RapidMiner Studio version 9.0 (Rapid Miner, Boston, MA, USA). Statistical tests were performed as indicated. Mann–Whitney U test was used as explained elsewhere^36^.

### IRB statement

As no humans were involved, IRB/ethics committee approval was waived.

## Supplementary Information

Supplementary Information.

## Data Availability

The datasets generated and/or analyzed during the current study, as well as a database of known organic substances (n ≈ 200), which was generated in-house, can be requested from co-author JIBB (email: joerg.baumbach@bbraun.com).
