# Supplementary material for: A proof of concept study for the differentiation of SARS-CoV-2, hCoV-NL63, and IAV-H1N1 in vitro cultures using ion mobility spectrometry
Source: Sci Rep. 2021 Oct 11;11:20143. doi: 10.1038/s41598-021-99742-7 (PMC8505652; doi:10.1038/s41598-021-99742-7)
Supplement: Supplementary file 1 — Supplementary Information. [file 41598_2021_99742_MOESM1_ESM.pdf]

Supplementary Information

A proof of concept study for the differentiation of SARS-CoV-2, human coronavirus-NL63, and influenza A virus H1N1 *in vitro* cultures using ion mobility spectrometry

Feuerherd, M.[1,2]*; Sippel, A.-K.[3]; Erber J.[4]; Baumbach, J.I.[3]; Schmid, R.M.[4]; Protzer, U.[1,2,5]; Voit, F.[4#]; Spinner, C.D.[4,5#]

[1] Institute of Virology, School of Medicine, Technical University of Munich, Munich, Germany

[2] Institute of Virology, Helmholtz Zentrum Munich, Munich, Germany

[3] B. Braun Melsungen AG, Branch Dortmund, Center of Competence Breath Analysis, BioMedicalCenter, Dortmund, Germany

[4] Department of Internal Medicine II, University Hospital Rechts der Isar, School of Medicine, Technical University of Munich, Munich, Germany

[5] German Center for Infection Research (DZIF), Munich Partner Site, Munich, Germany

[#] These authors contributed equally.

* Corresponding author:

Martin Feuerherd, M.Sc.
Institute of Virology
School of Medicine
Technical University of Munich
Munich, 81675
Germany
Phone: +49-89-4140-6822
E-mail: martin.feuerherd@tum.de

Principle of MCC-IMS

For a detailed review of the principles of MCC-IMS, please see Cumeras et al. [9, 10]. The basic principles are as follows. In an ion mobility spectrometer, ions are formed from analytes using ionization sources such as ß-radiation, UV lamps, electrical discharges, or chemical ionization. Unlike a mass spectrometer, all processes take place at ambient pressure, and no vacuum systems are required. Ions formed from the analyte are periodically introduced into a drift tube via a Bradbury–Nielsen shutter and are accelerated in an electric field in the direction of a Faraday plate. By measuring the time in the drift tube (defined as the drift time), the mobility of the ion at a known electric field strength can be calculated as the force for the movement. Thus, a continuous measurement of the electric current at the Faraday plate provides time-dependent signals and ideally different peaks for the ions of the different analytes. The peak height (signal intensity) is a measure of the concentration of a specific analyte. Detection limits in the ng/L to pg/L range (ppmv to pptv range) can be achieved. To improve the separation, gas chromatographic columns can be interposed between the sample loop and the inlet of the ionization chamber of the IMS. For direct sampling of volumes between a few microliters to approximately 10 mL without any further sample preparation, approximately 1,000 capillaries were connected in parallel (multi-capillary columns).

IMS-chromatograms are three-dimensional plots displaying retention time (MCC), drift time (IMS), and signal intensity. Drift time and retention time are characteristics of the ions formed from the analytes, whereas signal intensity corresponds to the concentration of the analytes. Using databases of drift time and retention time for different analytes allows the identification and quantification (after calibration) of substances.

The advantages and disadvantages of ion mobility spectrometry compared with other methods of breath gas analysis (e.g., mass spectrometry) are reviewed elsewhere [9, 10]. A major advantage of IMS is that the moisture of exhaled breath does not disturb the analysis because the water content of the sample is exploited for the ionization process (i.e., chemical ionization by proton transfer). IMS is characterized by high specificity in terms of ion size,

chemistry, and nature of the sample (due to the combination of drift time and ionization properties). These devices are lightweight, space-saving, and energy-efficient.

Mass spectrometry, on the other hand, provides greater information density, and larger databases are available. Surface acoustic wave sensors require even less space and power than those for IMS but have a high level of cross sensitivity. The minimum detection limit of FTIR is higher than that of IMS, but inherent problems such as optical interference and optical windows, especially in humid air, render it less suitable for breath analysis.

Supplementary Table S1: Peaks with the highest discriminatory power in pairwise comparisons can be assigned to organic substances. The top five peaks (according to the lowest Norm U rank sum values) differentiating every pair of infections are shown. The p-values of the Mann-Whitney U tests are provided. Up to three molecules that match a database of IMS-chromatograms of known chemical substances (20160426_SubstanzDbNIST_122_St_layer, B. Braun Melsungen AG, Center of Competence Breath Analysis, Dortmund, Germany (formerly B & S Analytik GmbH, Dortmund, Germany)) are listed in descending probability.

SARS-CoV-2 vs. hCoV-NL63

#	Peak	Norm U value	p-value	Possible detected metabolites
1	V46	0.059	< 0.0001	(+)-3-Carene; 3-octanone
2	V38	0.075	< 0.0001	Heptane, 2,2,4,6,6-pentamethyl-; 2-octanone
3	V84	0.077	< 0.0001	Decane
4	V66	0.110	< 0.0001	1,3-Cyclohexadiene, 1-methyl-4-(1-methylethyl)-; cyclohexene, 1-methyl-4-(1-methylethenyl)-, (S)-
5	V04	0.112	< 0.0001	2,3-butanediol; cyclohexanone

SARS-CoV-2 vs. IAV H1N1

#	Peak	Norm U value	p-value	Possible detected metabolites
1	V33	0.002	< 0.0001	1-pentanol; pentanal; 2-butanone
2	V12	0.004	< 0.0001	Acetophenone
3	V03	0.005	< 0.0001	Cyclohexanone, 5-methyl-2-(1-methylethyl)-
4	V49	0.019	< 0.0001	Naphthalene
5	V64	0.075	< 0.0001	no association

hCoV-NL63 vs. IAV H1N1

#	Peak	Norm U value	p-value	Possible detected metabolites
1	V33	0.000	< 0.0001	1-pentanol; pentanal; 2-butanone
2	V46	0.006	< 0.0001	(+)-3-Carene; 3-octanone
3	V38	0.016	< 0.0001	Heptane, 2,2,4,6,6-pentamethyl-; 2-octanone
4	V04	0.024	< 0.0001	2,3-Butanediol; cyclohexanone
5	V57	0.109	< 0.0001	Nonane

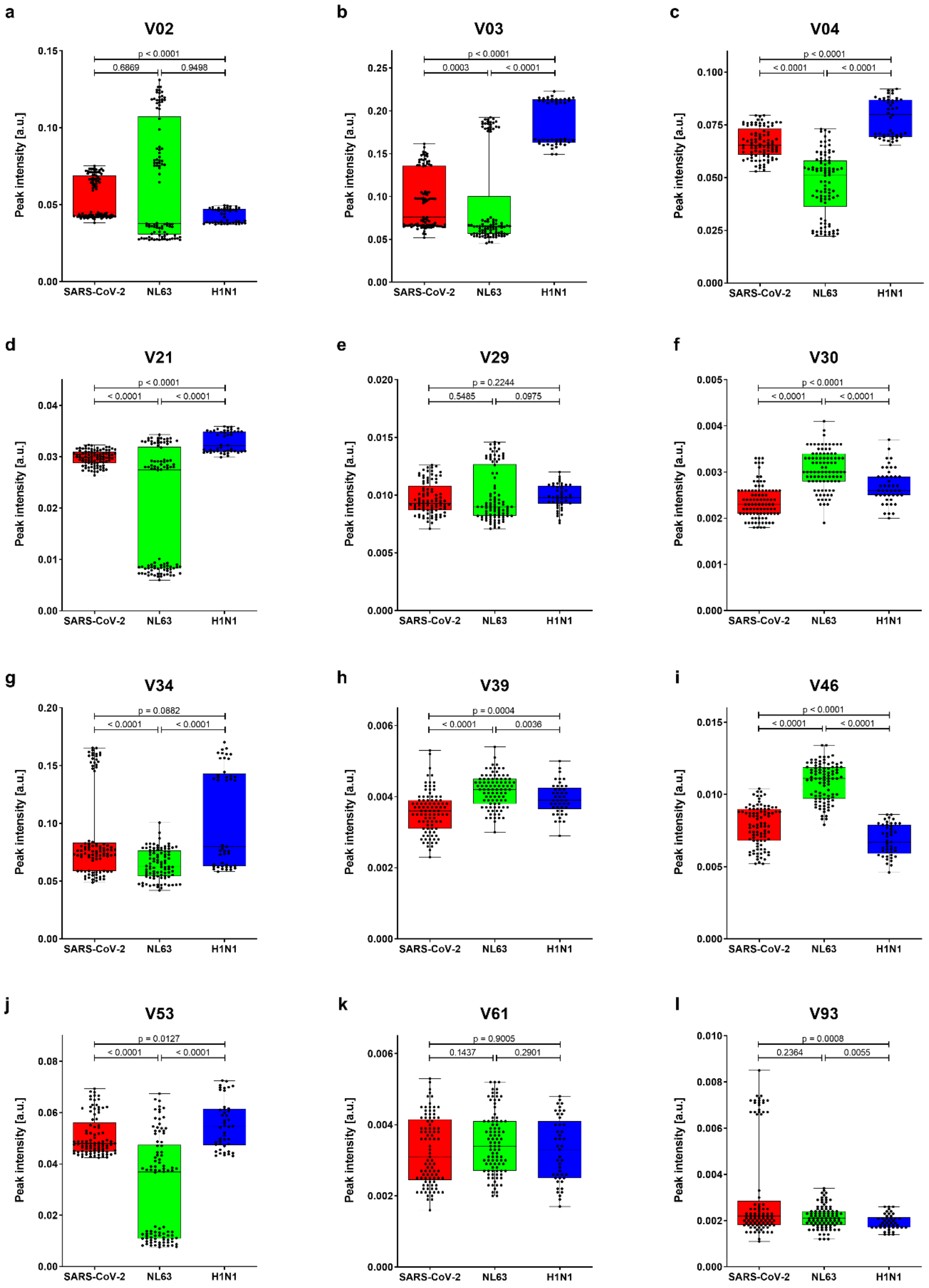

Supplementary Figure S2: Signal intensities of different peaks for SARS-CoV-2, human coronavirus NL63, and influenza A virus H1N1. Signal intensities in arbitrary units (a.u.) of peaks from headspace air samples of *in vitro* cultures of SARS-CoV-2 (red), hCoV-NL63 (green), and IAV-H1N1 (blue) infection are shown. Box-and-whisker plots represent the signal

intensity on day 3 post infection (p.i.) of peaks, which were used for the calculation of the decision tree (**a, b, j**) and forward selection (**c–i, k, l**). The intensity of each measurement is indicated by black dots (•). The central lines show the median, with colored boxes indicating interquartile ranges. The p-values were calculated with Mann-Whitney U test and are given in every subfigure. Data was visualized with GraphPad Prism 9.2.0 (https://www.graphpad.com/).

Supplementary Table S3: Differentiation power of peak V33 calculated using VisualNow for SARS-CoV-2, human coronavirus NL63, and influenza A virus H1N1 *in vitro* cultures. Note that the separation power is excellent for SARS-CoV-2 versus IAV-H1N1 and hCoV-NL63 versus IAV-H1N1, but not between SARS-CoV-2 and hCoV-NL63, which underlines the results reported in Figure 3.

	hCoV-NL63 vs. SARS-COV-2	IAV-H1N1 vs. SARS-CoV-2	hCoV-NL63 vs. IAV-H1N1
Peak	V33	V33	V33
Best direction	C1 > C2	C1 < C2	C1 > C2
Best threshold	0.019	0.017	0.017
Classified right	124	135	139
Classified wrong	63	3	0
True positive	66	44	94
False positive	35	2	0
True negative	58	91	45
False negative	28	1	0
Sensitivity	0.702	0.978	1.000
Specificity	0.624	0.978	1.000
Positive predictive value	0.653	0.957	1.000
Negative predictive value	0.674	0.989	1.000
a = sensitivity − (1 −specificity)	0.326	0.956	1.000
Accuracy	0.663	0.978	1.000
Mann–Whitney U	2423	10	0
z value from U	5.264	9.458	9.521
Significance level U (p-value)	< 0.001	<0.001	<0.001
Norm U value	0.277	0.002	0.000

Supplementary Table S4: Differentiation power of peak V02 calculated using VisualNow for SARS-CoV-2, human coronavirus NL63, and influenza A virus H1N1 *in vitro* cultures. Note that the separation power is excellent for SARS-CoV-2 versus IAV-H1N1 and hCoV-NL63 versus IAV-H1N1, but not between SARS-CoV-2 and hCoV-NL63, which underlines the results reported in Figure 3.

	hCoV-NL63 vs. SARS-COV-2	IAV-H1N1 vs. SARS-CoV-2	hCoV-NL63 vs. IAV-H1N1
Peak	V02	V02	V02
Best direction	C1 < C2	C1 < C2	C1 > C2
Best threshold	0.038	0.041	0.027
Classified right	141	113	94
Classified wrong	46	25	45
True positive	48	21	94
False positive	0	1	45
True negative	93	92	0
False negative	46	24	0
Sensitivity	0.511	0.467	1.000
Specificity	1.000	0.989	0.000
Positive predictive value	1.000	0.955	0.676
Negative predictive value	0.669	0.793	0
a = sensitivity − (1 −specificity)	0.511	0.456	0.000
Accuracy	0.754	0.819	0.676
Mann–Whitney U	4222	1112	2101
z value from U	0.403	4.453	0.063
Significance level U (p-value)	0.6869	< 0.001	0.9498
Norm U value	0.483	0.266	0.497

Supplementary Table S5: Differentiation power of peak V03 calculated using VisualNow for SARS-CoV-2, human coronavirus NL63, and influenza A virus H1N1 *in vitro* cultures. Note that the separation power is excellent for SARS-CoV-2 versus IAV-H1N1 and hCoV-NL63 versus IAV-H1N1, but not between SARS-CoV-2 and hCoV-NL63, which underlines the results reported in Figure 3.

	hCoV-NL63 vs. SARS-COV-2	IAV-H1N1 vs. SARS-CoV-2	hCoV-NL63 vs. IAV-H1N1
Peak	V03	V03	V03
Best direction	C1 < C2	C1 > C2	C1 < C2
Best threshold	0.064	0.156	0.149
Classified right	129	134	116
Classified wrong	58	4	23
True positive	43	43	71
False positive	7	2	0
True negative	86	91	45
False negative	51	2	23
Sensitivity	0.457	0.956	0.755
Specificity	0.925	0.978	1.000
Positive predictive value	0.860	0.956	1.000
Negative predictive value	0.628	0.978	0.662
a = sensitivity − (1 −specificity)	0.382	0.934	0.755
Accuracy	0.690	0.971	0.835
Mann–Whitney U	3039	21	552
z value from U	3.599	9.409	7.036
Significance level U (p-value)	< 0.001	< 0.001	< 0.001
Norm U value	0.348	0.005	0.130

Supplementary Table S6: Differentiation power of peak V53 calculated using VisualNow for SARS-CoV-2. human coronavirus NL63. and influenza A virus H1N1 *in vitro* cultures. Note that the separation power is excellent for SARS-CoV-2 versus IAV-H1N1 and hCoV-NL63 versus IAV-H1N1, but not between SARS-CoV-2 and hCoV-NL63, which underlines the results reported in Figure 3.

	hCoV-NL63 vs. SARS-COV-2	IAV-H1N1 vs. SARS-CoV-2	hCoV-NL63 vs. IAV-H1N1
Peak	V53	V53	V53
Best direction	C1 < C2	C1 > C2	C1 < C2
Best threshold	0.042	0.069	0.043
Classified right	157	101	110
Classified wrong	30	37	29
True positive	64	9	65
False positive	0	1	0
True negative	93	92	45
False negative	30	36	29
Sensitivity	0.681	0.200	0.691
Specificity	1.000	0.989	1.000
Positive predictive value	1.000	0.900	1.000
Negative predictive value	0.756	0.719	0.608
a = sensitivity − (1 −specificity)	0.681	0.189	0.691
Accuracy	0.840	0.732	0.791
Mann–Whitney U	1750	1544	645
z value from U	7.082	2.491	6.617
Significance level U (p-value)	< 0.001	< 0.05	< 0.001
Norm U value	0.200	0.369	0.152

Supplementary Table S7: Differentiation of viruses via forward selection including all measurement days (days 1–5). Day 1–5 p.i. headspace air samples of SARS-CoV-2, hCoV-NL63, and IAV- H1N1 cultures were evaluated with forward selection to determine the peaks, which can be used to achieve the best results in terms of testing reliability. The peaks chosen for the forward selection were V04, V21, V29, V30, V34, V39, V46, V61, and V93. Positive and negative predictive values, as well as sensitivity and specificity, were calculated.

		Infection					
		SARS-CoV-2	hCoV-NL63	IAV-H1N1	Total	Positive predictive value	Negative predictive value
Test	SARS-CoV-2	381	47	16	444	85.8%	89.5%
Test	hCoV-NL63	17	325	1	343	94.8%	92.5%
Test	IAV-H1N1	38	0	144	182	79.1%	97.8%
	Total	436	372	161			
	Sensitivity	87.4%	87.4%	89.4%			
	Specificity	88.2%	97.0%	95.3%			

Supplementary Figure S8: MCC-IMS chromatograms of headspace air samples of blank cell culture flasks, flasks with cell culture medium or flasks with cultured cells in comparison to SARS-CoV-2 infection (a) Exemplary IMS-chromatogram of a 10 mL headspace air sample collected after 24h of sampling from a blank flask, (b) a flask with cell culture medium, (c) a flask with cultured, uninfected cells and (d) a SARS-CoV-2 culture. Chromatograms were produced with VisualNow version 3.7 (permission granted as provided to the editor) and the axis labeling and legend were added with Affinity Designer 1.10 (https://affinity.serif.com/en-us/designer/).

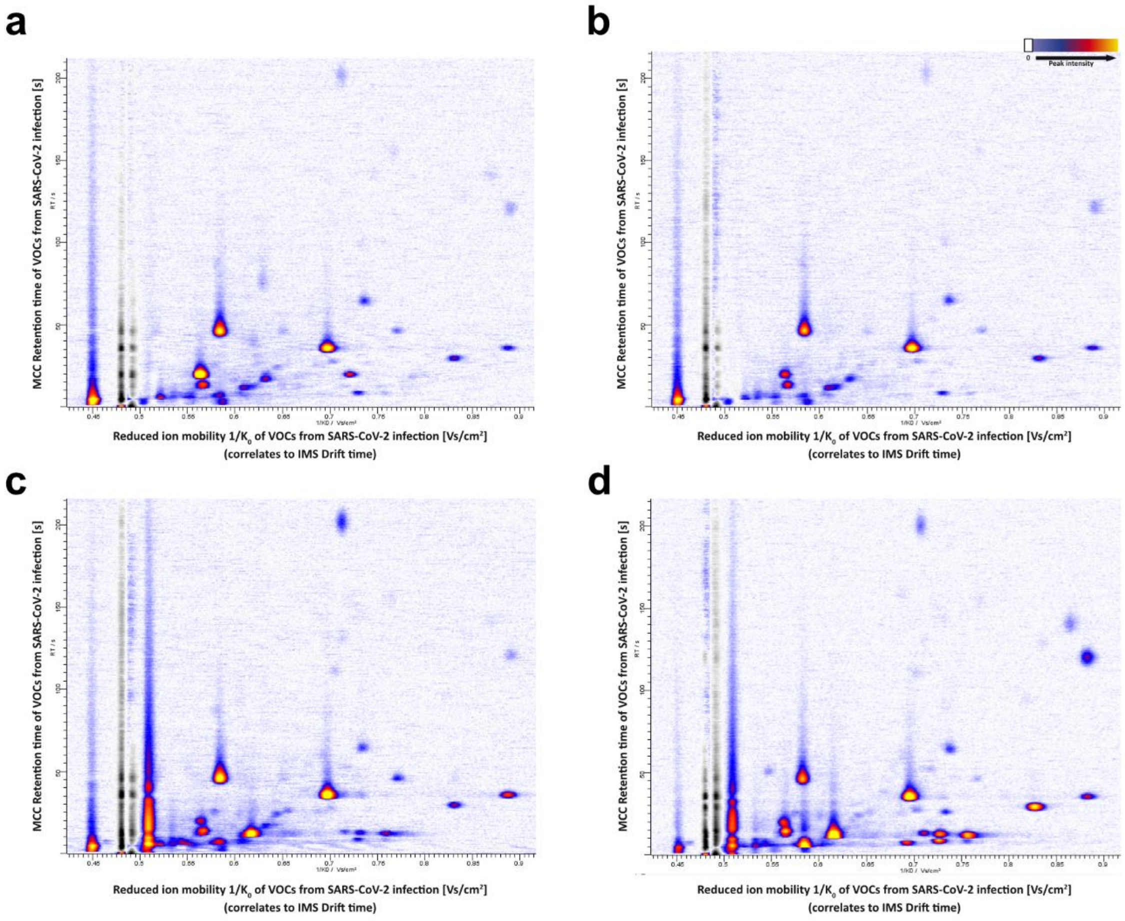

Supplementary Figure S9: MCC-IMS chromatograms of headspace air samples of SARS-CoV-2, hCoV-NL63 and IAV-H1N1 infection (a) Exemplary IMS-chromatogram of a 10 mL headspace air sample collected after 60h of sampling from a SARS-CoV-2 infection, (b) an hCoV-NL63 infection and (c) an IAV-H1N1 infection. Chromatograms were produced with VisualNow version 3.7 (permission granted as provided to the editor) and the axis labeling and legend were added with Affinity Designer 1.10 (https://affinity.serif.com/en-us/designer/).

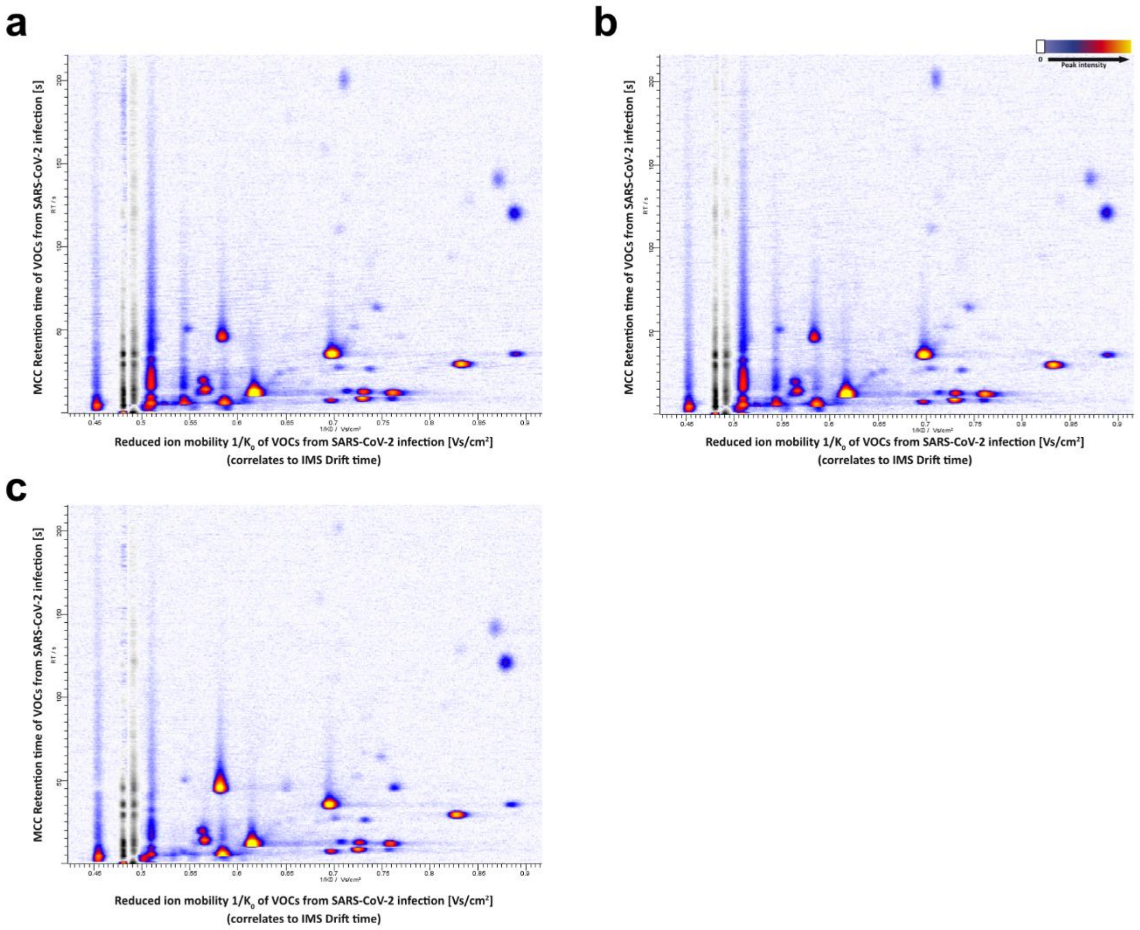

Supplementary Table S10: Parameters used for peak analysis via VisualNow

Base correction	True
Compensate RIP	True
Norm signal to RIP	True
Smooth	True
Median smooth	True
Align	True
Align k0	False
Align k0 to RIP	False
Amplify	12.0
Local baseline correction	False
